# The pharmacogenetics of *CYP2D6* and *CYP2C19* in a case series of antidepressant responses

**DOI:** 10.3389/fphar.2023.1080117

**Published:** 2023-02-21

**Authors:** Ping Siu Kee, Simran D. S. Maggo, Martin A. Kennedy, Paul K. L. Chin

**Affiliations:** ^1^ Department of Pathology and Biomedical Science, University of Otago, Christchurch, New Zealand; ^2^ Department of Pathology, Center for Personalized Medicine, Children’s Hospital Los Angeles, Los Angeles, CA, United States; ^3^ Department of Medicine, University of Otago, Christchurch, New Zealand; ^4^ Department of Clinical Pharmacology, Christchurch Hospital, Christchurch, New Zealand

**Keywords:** antidepressant, Adverse drug reaction, drug response, CYP2D6, CYP2C19, Psychiatry, clinical utility, pharmacogenetics

## Abstract

Pharmacogenetics has potential for optimizing use of psychotropics. *CYP2D6* and *CYP2C19* are two clinically relevant pharmacogenes in the prescribing of antidepressants. Using cases recruited from the Understanding Drug Reactions Using Genomic Sequencing (UDRUGS) study, we aimed to evaluate the clinical utility of genotyping *CYP2D6* and *CYP2C19* in antidepressant response. Genomic and clinical data for patients who were prescribed antidepressants for mental health disorders, and experienced adverse reactions (ADRs) or ineffectiveness, were extracted for analysis. Genotype-inferred phenotyping of *CYP2D6* and *CYP2C19* was carried out as per Clinical Pharmacogenetics Implementation Consortium (CPIC) guidelines. A total of 52 patients, predominantly New Zealand Europeans (85%) with a median age (range) of 36 years (15–73), were eligible for analysis. Thirty-one (60%) reported ADRs, 11 (21%) ineffectiveness, and 10 (19%) reported both. There were 19 CYP2C19 NMs, 15 IMs, 16 RMs, one PM and one UM. For CYP2D6, there were 22 NMs, 22 IMs, four PMs, three UMs, and one indeterminate. CPIC assigned a level to each gene-drug pair based on curated genotype-to-phenotype evidence. We analyzed a subgroup of 45 cases, inclusive of response type (ADRs/ineffectiveness). Seventy-nine (N = 37 for *CYP2D6*, N = 42 for *CYP2C19*) gene-drug/antidepressant-response pairs with CPIC evidence levels of A, A/B, or B were identified. Pairs were assigned as ‘actionable’ if the CYP phenotypes potentially contributed to the observed response. We observed actionability in 41% (15/37) of *CYP2D6*-antidepressant-response pairs and 36% (15/42) of *CYP2C19*-antidepressant-response pairs. In this cohort, *CYP2D6* and *CYP2C19* genotypes were actionable for a total of 38% pairs, consisting of 48% in relation to ADRs and 21% in relation to drug ineffectiveness.

## 1 Introduction

Heterogeneity in drug response is a well-recognized challenge in mental health. In the management of depression, the average response rate reported for antidepressants ranged from 42% to 53% (Cipriani et al., 2018a; Cipriani et al., 2018b), while nearly 50% of patients experienced adverse reactions (ADRs) during treatment (Braund et al., 2021). ADRs and ineffectiveness are a major contributor towards poor adherence and discontinuation of antidepressants (Marasine and Sankhi, 2021).

Pharmacogenetics is a promising clinical tool in antidepressant prescribing, which aims to improve depression remission rates and minimize ADRs (Bousman et al., 2017). Systematic reviews and meta-analyses have supported the clinical utility of pharmacogenetic tests in depression management with significant improvement on patient outcomes observed for pharmacogenetics-guided groups (Arnone et al., 2023; Brown et al., 2020; Brown et al., 2022). Further, the recent Pre-emptive Pharmacogenomic Testing for Preventing Adverse Drug Reactions (PREPARE) randomised controlled trial, which included antidepressant-gene combinations, showed that genotype-guided prescribing reduced the incidence of clinically relevant ADRs by 30% (Swen et al., 2023).

Genetic variants of *CYP2D6* and *CYP2C19* are most clinically relevant to the pharmacokinetics of antidepressants. Literature investigating the association between *CYP2D6/CYP2C19* genotypes and antidepressant response is extensive (Rosenblat et al., 2018; Bousman et al., 2019; Solomon et al., 2019), and its clinical implementation is advancing (van Westrhenen et al., 2020). The Clinical Pharmacogenetics Implementation Consortium (CPIC) has published *CYP2D6* and *CYP2C19* prescribing guidelines for two antidepressant classes (tricyclic antidepressants (TCAs) and selective serotonin reuptake inhibitors (SSRIs)) (Hicks et al., 2015; Hicks et al., 2017), with the updated guideline in review for publication. For each gene-drug pair, CPIC has reviewed the available evidence linking genotype to phenotype, and assigned a level of evidence (A, A/B, B, B/C, C, C/D, or D). Pharmacogenetics-guided treatments for antidepressants assigned with CPIC evidence levels A and B have been reported to be either cost-effective or cost-saving (Morris et al., 2022).

For gene-drug pairs with level A, the genetic information *should* be used to change prescribing of affected drug, while for B, the genetic information *could* be used to change prescribing of the affected drug because alternative therapies or dosing are extremely likely to be as effective and as safe as non-genetically based dosing. Currently, only gene-drug pairs with level A and B are sufficient for at least one prescribing action to be recommended. For level A/B, full evidence review is to be undertaken by CPIC, with preliminary review indicating a definitive CPIC level of either A or B (Clinical Pharmacogenetics Implementation Consortium, 2021b).

Understanding Adverse Drug Reactions using Genomic Sequencing (UDRUGS) is an ongoing project by our laboratory, which recruits patients who have experienced ADRs or drug ineffectiveness (Maggo et al., 2017). In addition to establishing a DNA bank linked to the clinical information of this cohort, UDRUGS also aims to study the role of genetic variations in known pharmacogenes that may contribute to the observed phenotypes. Recruited cases are mainly referred by healthcare practitioners, with established ADRs or ineffectiveness (treatment failure) phenotypes. In this case series report, we used cases recruited into UDRUGS to examine the explanatory role of *CYP2D6* and *CYP2C19* pharmacogenetics in responses associated with CPIC evidence levels A, A/B, or B-assigned gene-antidepressant pairs.

## 2 Materials and methods

The Understanding Adverse Drug Reactions using Genomic Sequencing (UDRUGS) study (Maggo et al., 2017) received ethical approval from the New Zealand (NZ) Health and Disability Ethics Committees (HDEC URA/11/11/065). This retrospective case series report aims to evaluate the clinical utility of genotyping *CYP2D6* and *CYP2C19* in antidepressant response.

### 2.1 Study cases

The recruitment and sampling of UDRUGS cases (Maggo et al., 2017), and deoxyribonucleic acid (DNA) extraction were as previously described (Miller et al., 1988; Maggo et al., 2019b). Screening was carried out on UDRUGS cases recruited during May 2019 - December 2021. The case inclusion criteria were patients who were prescribed antidepressants for mental health disorders, and experienced ADRs or ineffectiveness, and cases with complete *CYP2D6* and *CYP2C19* genotype results for analysis. The genomic and clinical information for these cases were extracted and analysed *via* two approaches as described in subsequent Section 2.3.

### 2.2 Existing data on genotype, haplotype, and phenotype

The genotypes of *CYP2D6* and *CYP2C19* were available for each of the identified UDRUGS cases, informed by previous screening of selected genetic variants. The genetic analysis for these pharmacogenes was carried out as previously reported (Maggo et al., 2019b; Kee et al., 2022). Briefly, gene regions of interest were amplified by polymerase chain reactions (PCR) and subsequently subjected to Sanger sequencing, run using BigDye^®^ Terminator v3.1 on a 3130XL Genetic Analyser. Generated chromatograms were aligned with reference sequences for genotyping, *via* Geneious Prime Version 2020.1 (Biomatters Ltd. Auckland, NZ). *1 allele was assigned when no variations were observed. For *CYP2C19*, *2 (rs4244285), *3 (rs4986893), and *17 (rs12248560) alleles were genotyped. These are the first tier of *CYP2C19* alleles recommended in clinical genotyping (Pratt et al., 2018). Unlike *CYP2C19*, the screening of *CYP2D6* involved the whole 6.6 kb gene. This approach was preferred due to its polymorphic nature. To date, Pharmacogene Variation Consortium (PharmVar) has listed over 170 *CYP2D6* star alleles (Gaedigk et al., 2018; Gaedigk et al., 2021). Alleles were assigned according to the PharmVar nomenclature system (Gaedigk et al., 2018). Based on the patient’s genotype, cases were assigned a phenotype according to CPIC guidelines (Caudle et al., 2020; Crews et al., 2021; Lima et al., 2021).

### 2.3 Case analysis

The methodology and study flow is as illustrated in Supplementary Figure S1.

#### 2.3.1 Drug/antidepressant-response pairs

This is defined as the number of cases reporting any negative response (ADRs or ineffectiveness) with each antidepressant. For example, a case which documented both ADRs and ineffectiveness with the use of citalopram is calculated as one response with the respective antidepressant.

#### 2.3.2 Gene-drug/antidepressant-response pairs

The list of antidepressants for which CPIC has assigned an evidence level of A, A/B, or B for *CYP2D6* or *CYP2C19* is shown in Supplementary Table S1 (Clinical Pharmacogenetics Implementation Consortium, 2021a). From the eligible UDRUGS cases, gene-drug-response pairs were extracted for analysis. This observation also considered the type of negative response. For example, a case which documented both ADRs and ineffectiveness with the use of citalopram is calculated as two observation pairs. This is presented as ‘*CYP2D6/CYP2C19*-antidepressant-response pairs’.

Identified *CYP2D6/CYP2C19*-antidepressant-response pairs were categorized into either “actionable” or “non-actionable”. The proportion of actionable and non-actionable *CYP2D6/CYP2C19*-antidepressant-response pairs was assessed for 1) Antidepressant drug class, 2) Individual antidepressant, 3) CPIC evidence levels A, A/B, and B, 4) *CYP2D6* and *CYP2C19* pharmacogenes, and 5) Drug response phenotypes (ADRs and ineffectiveness).

#### 2.3.3 Definition of “actionability”

The assignment was informed by critically assessing the available clinical and genetic data for the association between 1) genotype-inferred phenotypes, 2) drug exposure, and 3) drug response events.

Compared with normal metabolizer (NMs) phenotypes, a reduced metabolism rate is predicted in intermediate metabolizers (IMs) and poor metabolizers (PMs), while, an increased rate of metabolism is predicted in rapid metabolizers (RMs) and ultrarapid metabolizers (UMs). For drug exposure, it is predicted to be higher in IMs and PMs, and lower in RMs and UMs. In this cohort, two response phenotypes were analyzed: ADRs and ineffectiveness. High and low drug exposure levels are predicted to predispose to the risk of ADRs and ineffectiveness, respectively. “Ineffectiveness” included partial response, poor response, diminished response, or an “unusually” high dose requirement (as reported by the healthcare practitioner referring the case).

Pairs with a clear association between these three aspects are assigned as “actionable”. For example, for a medicine where the parent drug is active and its metabolites are inactive, the decreased or absent *CYP2D6* and/or *CYP2C19* metabolic capacity of a genetically-derived IM or PM could result in a higher drug exposure, increasing the risk of ADRs, or *vice versa* for RMs and UMs with the ineffectiveness phenotypes.

### 2.4 Statistical analyses

Binomial proportion confidence intervals, using the Wilson score interval method, were used to calculate all proportions and confidence intervals, using OpenEpi, a free and open source statistical software (Dean et al., 2013).

## 3 Results

### 3.1 Case identification and analysis

A total of 205 UDRUGS cases recruited between May 2019 to December 2021 were assessed. Of these, 52 eligible cases experienced ADRs or ineffectiveness with one or more antidepressants indicated for mental health conditions, and with complete *CYP2D6* and *CYP2C19* genotypes. The majority of cases (49) were referred by healthcare practitioners (including doctors and pharmacists), while three were self-referred.

A total of 111 antidepressant-response pairs were identified from 52 cases. Of the 111 antidepressant-response pairs observed, 58 (53%) involved SSRIs, 17 (15%) TCAs and 17 (15%) selective norepinephrine reuptake inhibitors (SNRIs). Others included 4 (4%) monoamine oxidase inhibitors (MAO-I), 13 (12%) atypical agents (bupropion and mirtazapine), and two (2%) were unspecified. The highest number of responses documented involved the use of fluoxetine (N = 22), followed by venlafaxine (N = 17) and sertraline (N = 15), while the recorded number of antidepressant-response pairs was comparable for nortriptyline (N = 12), escitalopram (N = 12) and mirtazapine (N = 11). Table 1 summarizes case demographic information and a breakdown of antidepressant-response pairs.

**TABLE 1:** Summary of the recruited cases (median, range).

Variables	Total cases (N = 52)
Sex (count): Female	40 (77%)
Age (years)	36 (15–73)
Ethnicity	
• European	44 (84.6%)
• Mixed European descent (New Zealand Māori, Asian and Pasifika)	4 (7.7%)
• New Zealand Māori	2 (3.8%)
• Asian	1 (1.9%)
• Middle Eastern	1 (2%)
Cases associated with adverse reactions only	31 (60%)
Cases associated with ineffectiveness only	11 (21%)
Cases associated with both adverse reactions and ineffectiveness	10 (19%)
Antidepressant-response pairs	Total pairs (N = 111)
Tricyclic Antidepressants (TCAs)	
• Amitriptyline	3 (3%)
• Clomipramine	2 (2%)
• Nortriptyline	12 (11%)
Selective serotonin reuptake inhibitors (SSRIs)	
• Citalopram	6 (5%)
• Escitalopram	12 (11%)
• Fluoxetine	22 (20%)
• Paroxetine	2 (2%)
• Sertraline	15 (14%)
• Medications were not specified	1 (1%)
Selective norepinephrine reuptake inhibitors (SNRIs)	
• Venlafaxine	17 (15%)
Monoamine-oxidase inhibitors (MAO-I)	
• Moclobemide	2 (2%)
• Tranylcypromine	2 (2%)
Atypical antidepressants	
• Mirtazapine	11 (10%)
• Bupropion	2 (2%)
Type of antidepressants were not specified by healthcare practitioners or patients	2 (2%)

The documented ADRs covered a wide range of symptoms, mainly alteration of mental state (e.g., agitation, manic episode, paranoid), autonomic effects (e.g., diarrhoea, heart palpitation, diaphoresis), and neuromuscular effects (e.g., dystonia, stiffness). For efficacy, the majority of the cases either did not respond to or response diminished over time. These responses were documented across a range of doses and the time of event onset ranged from hours to months of being on treatment. The details of individual cases can be found in Supplementary Table S2.

### 3.2 Genotypes and phenotypes of the extracted cases

Of the 52 cases, there were 22 (42%) CYP2D6 NMs, 22 (42%) IMs, four (8%) PMs, three (6%) UMs, and one (2%) indeterminate case. *CYP2D6* duplications were detected in cases B35 (*1/*1xN), B38 (*1/*35xN or *1xN/*35), and B52 (*1/*27xN or *1xN/*27). Since *1, *35, and *27 are normal function alleles, the predicted phenotype was UM regardless of which allele is duplicated. The phenotype of sample B2 (*4/*32) was indeterminate due to the uncertain function of the *32 allele (Phasing was confirmed through long read Nanopore sequencing; protocols as described in (Hitchman et al., 2022)). With the presence of a non-functional *4 allele, B2 is expected to be either IM or PM. For CYP2C19, there was one (2%) UM, 16 (31%) RMs, 19 (37%) NMs, 15 (29%) IMs, and one (2%) PM.

The genotypes, inferred CYP2D6 and CYP2C19 phenotypes, and reported antidepressants and responses for each case can be found in Table 2.

**TABLE 2:** Genotypes and phenotypes of recruited cases (N = 52).

Case	^a^Types of response	Antidepressants	^b^ *CYP2D6*	^b^ *CYP2C19*
B1	ADR	Amitriptyline	*9/*41 (IM)	*1/*17 (RM)
B2	ADR	Amitriptyline, sertraline, bupropion	*4/*32 ^c^ (Indeterminate)	*1/*3 (IM)
B3	ADR	Fluoxetine	*1/*4 (IM)	*1/*17 (RM)
B4	ADR	Amitriptyline	*2/*41 (NM)	*1/*17 (RM)
B5	ADR, IE	Escitalopram, bupropion	*35/*41 (NM)	*1/*1 (NM)
B6	ADR, IE	Fluoxetine, nortriptyline	*1/*9 (NM)	*1/*17 (RM)
B7	IE	Paroxetine, nortriptyline, sertraline	*9/*9 (IM)	*1/*1 (NM)
B8	ADR, IE	Clomipramine, moclobemide, buspirone, nortriptyline, fluoxetine, escitalopram	*2/*5 (IM)	*1/*17 (RM)
B9	ADR	Sertraline	*4/*4 (PM)	*1/*17 (RM)
B10	ADR	Venlafaxine, nortriptyline	*2/*4 (IM)	*1/*17 (RM)
B11	ADR, IE	Citalopram, fluoxetine	*4/*41 (IM)	*1/*1 (NM)
B12	ADR	Fluoxetine, venlafaxine, sertraline	*2/*2 (NM)	*1/*2 (IM)
B13	ADR	Mirtazapine, fluoxetine	*2/*4 (IM)	*1/*1 (NM)
B14	ADR	Medications were not specified	*1/*1 (NM)	*1/*2 (IM)
B15	IE	Fluoxetine, sertraline, bupropion, venlafaxine, mirtazapine	*1/*9 (NM)	*1/*2 (IM)
B16	IE	Tranylcypromine, nortriptyline, fluoxetine, venlafaxine	*1/*2 (NM)	*1/*1 (NM)
B17	ADR	Mirtazapine	*2/*2 (NM)	*1/*2 (IM)
B18	IE	Venlafaxine, mirtazapine	*2/*4 (IM)	*1/*1 (NM)
B19	ADR	Paroxetine, escitalopram, fluoxetine, mirtazapine	*1/*4 (IM)	*1/*2 (IM)
B20	ADR, IE	Venlafaxine, mirtazapine, nortriptyline, escitalopram	*4/*9 (IM)	*1/*2 (IM)
B21	ADR	Venlafaxine	*1/*10 (NM)	*1/*1 (NM)
B22	ADR, IE	Venlafaxine, escitalopram, sertraline	*1/*9 (NM)	*1/*1 (NM)
B23	ADR	Fluoxetine	*1/*4 (IM)	*1/*1 (NM)
B24	ADR	Nortriptyline, venlafaxine, mirtazapine	*1/*1 (NM)	*1/*17 (RM)
B25	ADR	Fluoxetine	*2/*9 (NM)	*1/*1 (NM)
B26	ADR	Fluoxetine, sertraline	*1/*41 (NM)	*1/*1 (NM)
B27	ADR	Citalopram, fluoxetine	*1/*1 (NM)	*2/*17 (IM)
B28	IE	Fluoxetine, sertraline	*1/*1 (NM)	*2/*2 (PM)
B29	ADR, IE	Venlafaxine, escitalopram	*1/*1 (NM)	*1/*1 (NM)
B30	ADR, IE	Fluoxetine, mirtazapine, escitalopram, venlafaxine	*4/*10 (IM)	*1/*2 (IM)
B31	ADR	Fluoxetine, sertraline, moclobemide	*1/*1 (NM)	*1/*17 (RM)
B32	ADR	Venlafaxine, citalopram	*4/*41 (IM)	*1/*2 (IM)
B33	ADR	Nortriptyline	*1/*4 (IM)	*1/*2 (IM)
B34	ADR, IE	Fluoxetine, citalopram, nortriptyline, escitalopram, venlafaxine, tranylcypromine	*1/*1 (NM)	*1/*17 (RM)
B35	IE	Fluoxetine	*1/*1x ^d^ *N* (UM)	*1/*1 (NM)
B36	ADR	Fluoxetine, sertraline	*2/*4 (IM)	*1/*1 (NM)
B37	ADR	Nortriptyline	*4/*4 (PM)	*1/*2 (IM)
B38	ADR	Escitalopram, venlafaxine	*1/*35x ^d^ *N* or *1x ^d^ N/*35 (UM)	*1/*1 (NM)
B39	IE	Escitalopram	*2/*3 (IM)	*1/*1 (NM)
B40	ADR	Sertraline, mirtazapine	*1/*2 (NM)	*1/*1 (NM)
B41	ADR	Sertraline	*4/*4 (PM)	*1/*17 (RM)
B42	ADR	Nortriptyline, venlafaxine	*1/*1 (NM)	*1/*17 (RM)
B43	IE	Fluoxetine, clomipramine	*5/*35 (IM)	*2/*17 (IM)
B44	ADR	Sertraline	*1/*1 (NM)	*17/*17 (UM)
B45	ADR	Selective serotonin reuptake inhibitors (medications were not specified)	*1/*5 (IM)	*1/*1 (NM)
B46	IE	Nortriptyline, venlafaxine, mirtazapine	*2/*4 (IM)	*1/*17 (RM)
B47	IE	Medications were not specified	*4/*4 (PM)	*2/*17 (IM)
B48	IE	Fluoxetine, sertraline	*1/*4 (IM)	*1/*17 (RM)
B49	ADR	Citalopram	*1/*4 (IM)	*1/*17 (RM)
B50	ADR, IE	Escitalopram, fluoxetine, citalopram, mirtazapine	*4/*33 (IM)	*2/*17 (IM)
B51	ADR	Venlafaxine, selective serotonin reuptake inhibitors except escitalopram which is tolerable (medications were not specified)	*1/*10 (NM)	*1/*17 (RM)
B52	ADR	Sertraline, escitalopram	*1/*27x ^d^ *N* or *1x ^d^ N/*27 (UM)	*1/*1 (NM)

^a^ ADR: adverse reactions, IE: ineffectiveness.

^b^ UM: ultrarapid metabolizer, RM: rapid metabolizer, NM: normal metabolizer, IM: intermediate metabolizer, PM: poor metabolizer.

^c^ The function of the CYP2D6*32 allele is uncertain.

^d^ “N” indicates the presence of a gene duplication or multiplication (gene copy number unknown) alleles.

### 3.3 Characterization of *CYP2D6/CYP2C19*-antidepressant-response pairs

Of the 52 cases, seven were excluded from this analysis for two reasons. First, the antidepressants documented have not been reviewed by CPIC, or have been assigned a lower level of evidence, specifically the drugs fluoxetine (CPIC evidence level C) and mirtazapine (CPIC evidence level B/C) (Clinical Pharmacogenetics Implementation Consortium, 2021a). Cases excluded for this reason were B3, B13, and B17. Second, specific antidepressants were not documented for B14, B45, B47, and B51.

Of the remaining 45 cases, 26 (57%) were CYP2D6 non-NMs and 28 (62%) were CYP2C19 non-NMs. A total of 79 gene-drug-response pairs were identified involving the antidepressant drug classes SSRIs, TCAs, and SNRIs. The specific drugs included nortriptyline, amitriptyline, clomipramine, paroxetine, sertraline, citalopram, escitalopram, and venlafaxine.

Table 3 lists the analyzed gene-drug-response pairs and their actionability based on *CYP2D6* and *CYP2C19* genotypes. The majority of the assignments were informed by the association between drug exposure and the metabolic rate predicted from genotype-inferred phenotypes, except for 1) Case B1 and B4 (*CYP2C19*-Amitriptyline-ADR pair), 2) Case B8 (*CYP2C19*-Clomipramine-ADR pair), and 3) Case B18 and B46 (*CYP2D6*-Venlafaxine-Ineffectiveness pair), where the associations were complex, mainly for two reasons. First, both the parent compound and metabolite are pharmacologically active (e.g., amitriptyline, clomipramine, and venlafaxine). Second, the role of *CYP2D6* and *CYP2C19* in the metabolic pathways is equally important, with similar CPIC evidence levels assigned (e.g., amitriptyline and clomipramine). These cases are described in further detail below.

**TABLE 3:** Analysed gene-antidepressant-response pairs and actionability of *CYP2D6* and *CYP2C19* pharmacogenetics (N = 79).

No	Case	Genotype-inferred phenotype		^a^Gene-antidepressant-response pairs	*CYP2D6* genotype		*CYP2C19* genotype	
		*CYP2D6*	*CYP2C19*		Actionable	Non-actionable	Actionable	Non-actionable
1	B1	Intermediate	Rapid	*CYP2D6*-Amitriptyline-ADR	√		N/A	N/A
2				*CYP2C19*-Amitriptyline-ADR	N/A	N/A	√	
3	B2	Intermediate or poor	Intermediate	*CYP2D6*-Amitriptyline-ADR	√		N/A	N/A
4				*CYP2C19*-Amitriptyline-ADR	N/A	N/A	√	
5				*CYP2C19*-Sertraline-ADR	N/A	N/A	√	
6	B4	Normal	Rapid	*CYP2D6*-Amitriptyline-ADR		√	N/A	N/A
7				*CYP2C19*-Amitriptyline-ADR	N/A	N/A	√	
8	B5	Normal	Normal	*CYP2C19*- Escitalopram-ADR	N/A	N/A		√
9				*CYP2C19*-Escitalopram-IE	N/A	N/A		√
10	B6	Normal	Rapid	*CYP2D6*-Nortriptyline-ADR		√	N/A	N/A
11	B7	Normal	Normal	*CYP2D6*-Nortriptyline-IE		√	N/A	N/A
12				*CYP2D6*-Paroxetine-IE		√	N/A	N/A
13				*CYP2C19*-Sertraline-IE	N/A	N/A		√
14	B8	Intermediate	Rapid	*CYP2D6*-Clomipramine-ADR	√		N/A	N/A
15				*CYP2D6*-Clomipramine-IE		√	N/A	N/A
16				*CYP2C19*-Clomipramine-ADR	N/A	N/A	√	
17				*CYP2C19*-Clomipramine-IE	N/A	N/A		√
18				*CYP2D6*-Nortriptyline-ADR	√		N/A	N/A
19				*CYP2D6*-Nortriptyline-IE		√	N/A	N/A
20				*CYP2C19*-Escitalopram-ADR	N/A	N/A		√
21				*CYP2C19*-Escitalopram-IE	N/A	N/A	√	
22	B9	Poor	Rapid	*CYP2C19*-Sertraline-ADR	N/A	N/A		√
23	B10	Intermediate	Rapid	*CYP2D6*-Nortriptyline-ADR	√		N/A	N/A
24				*CYP2D6*-Venlafaxine-ADR	√		N/A	N/A
25	B11	Intermediate	Normal	*CYP2C19*-Citalopram-ADR	N/A	N/A		√
26	B12	Normal	Intermediate	*CYP2D6*-Venlafaxine-ADR		√	N/A	N/A
27				*CYP2C19*-Sertraline-ADR	N/A	N/A	√	
28	B15	Normal	Intermediate	*CYP2D6-*Venlafaxine-IE		√	N/A	N/A
29				*CYP2C19*-Sertraline-IE	N/A	N/A		√
30	B16	Normal	Normal	*CYP2D6-*Venlafaxine-IE		√	N/A	N/A
31				*CYP2D6*-Nortriptyline-IE		√	N/A	N/A
32	B18	Intermediate	Normal	*CYP2D6-*Venlafaxine-IE	√		N/A	N/A
33	B19	Intermediate	Intermediate	*CYP2D6*-Paroxetine-ADR	√		N/A	N/A
34				*CYP2C19*-Escitalopram-ADR	N/A	N/A	√	
35	B20	Intermediate	Intermediate	*CYP2D6*-Venlafaxine-ADR	√		N/A	N/A
36				*CYP2D6*-Nortriptyline-ADR	√		N/A	N/A
37				*CYP2C19*-Escitalopram-IE	N/A	N/A		√
38	B21	Normal	Normal	*CYP2D6*-Venlafaxine-ADR		√	N/A	N/A
39	B22	Normal	Normal	*CYP2D6*-Venlafaxine-ADR		√	N/A	N/A
40				*CYP2C19*-Escitalopram-IE	N/A	N/A		√
41				*CYP2C19*-Sertraline-IE	N/A	N/A		√
42	B24	Normal	Rapid	*CYP2D6*-Venlafaxine-ADR		√	N/A	N/A
43				*CYP2D6-*Nortriptyline-ADR		√	N/A	N/A
44	B26	Normal	Normal	*CYP2C19*-Sertraline-ADR	N/A	N/A		√
45	B27	Normal	Intermediate	*CYP2C19*-Citalopram-ADR	N/A	N/A	√	
46	B28	Normal	Poor	*CYP2C19-*Sertraline-IE	N/A	N/A		√
47	B29	Normal	Normal	*CYP2D6*-Venlafaxine-IE		√	N/A	N/A
48				*CYP2C19*-Escitalopram-ADR	N/A	N/A		√
49	B30	Intermediate	Intermediate	*CYP2D6*-Venlafaxine-ADR	√		N/A	N/A
50				*CYP2C19*-Escitalopram-ADR	N/A	N/A	√	
51				*CYP2C19*-Escitalopram-IE	N/A	N/A		√
52	B31	Normal	Rapid	*CYP2C19*-Sertraline-ADR	N/A	N/A		√
53	B32	Intermediate	Intermediate	*CYP2D6-*Venlafaxine-ADR	√		N/A	N/A
54				*CYP2C19*-Citalopram-ADR	N/A	N/A	√	
55	B33	Intermediate	Intermediate	*CYP2D6*-Nortriptyline-ADR	√		N/A	N/A
56	B34	Normal	Rapid	*CYP2D6*-Nortriptyline-ADR		√	N/A	N/A
57				*CYP2D6*-Venlafaxine-IE		√	N/A	N/A
58				*CYP2C19*-Citalopram-IE	N/A	N/A	√	
59				*CYP2C19*-Escitalopram-IE	N/A	N/A	√	
60	B36	Intermediate	Normal	*CYP2C19*-Sertraline-ADR	N/A	N/A		√
61	B37	Poor	Intermediate	*CYP2D6*-Nortriptyline-ADR	√		N/A	N/A
61	B38	Ultrarapid	Normal	*CYP2D6*-Venlafaxine-ADR		√	N/A	N/A
62				*CYP2C19*-Escitalopram-ADR	N/A	N/A		√
63	B39	Intermediate	Normal	*CYP2C19*-Escitalopram-IE	N/A	N/A		√
64	B40	Normal	Normal	*CYP2C19*-Sertraline-ADR	N/A	N/A		√
65	B41	Poor	Rapid	*CYP2C19*-Sertraline-ADR	N/A	N/A		√
66	B42	Normal	Rapid	*CYP2D6*-Nortriptyline-ADR		√	N/A	N/A
67				*CYP2D6*-Venlafaxine-ADR		√	N/A	N/A
68	B43	Intermediate	Intermediate	*CYP2D6*-Clomipramine-IE		√	N/A	N/A
69				*CYP2C19*-Clomipramine-IE	N/A	N/A		√
70	B44	Normal	Ultrarapid	*CYP2C19*-Sertraline-ADR	N/A	N/A		√
71	B46	Intermediate	Rapid	*CYP2D6*-Nortriptyline-IE		√	N/A	N/A
73				*CYP2D6*-Venlafaxine-IE	√		N/A	N/A
74	B48	Intermediate	Rapid	*CYP2C19*-Sertraline-IE	N/A	N/A	√	
75	B49	Intermediate	Rapid	*CYP2C19*-Citalopram-ADR	N/A	N/A		√
76	B50	Intermediate	Intermediate	*CYP2C19*-Citalopram-IE	N/A	N/A		√
77				*CYP2C19*-Escitalopram-ADR	N/A	N/A	√	
78	B52	Ultrarapid	Normal	*CYP2C19*-Sertraline-ADR	N/A	N/A		√
79				*CYP2C19*-Escitalopram-ADR	N/A	N/A		√

^a^ ADR: adverse reactions, IE: ineffectiveness.

#### 3.3.1 B1 and B4 (*CYP2C19*-amitriptyline-ADR pair)

B1, a CYP2D6 IM and CYP2C19 RM. B4, a CYP2D6 NM and CYP2C19 RM. The rapid metabolism of CYP2C19 is expected to increase the conversion of amitriptyline to nortriptyline, which itself is pharmacologically active. When compared with amitriptyline, nortriptyline was five times more potent in inhibiting noradrenaline with less affinity towards other post-synaptic receptors, thus less ADRs and better tolerability (Gillman, 2007). This may explain the reported mechanism-related reactions (nightmare and severe exhaustion) in B4, which are thought to be caused by a shift of neurotransmitter concentrations, rather than effects associated with the non-selective binding on other post-synaptic receptors.

#### 3.3.2 B8 (*CYP2C19*-clomipramine-ADR pair)

B8, a CYP2D6 IM and CYP2C19 RM. The rapid metabolism of CYP2C19 is expected to increase the conversion of clomipramine to desmethyl-clomipramine, an active metabolite (Balant-Gorgia et al., 1991), which is further hydroxylated by CYP2D6. The association between *CYP2D*6 genotypes and the total clearance of clomipramine and hydroxylation indexes has been reported (Nielsen et al., 1994). Clinical case studies have also observed higher levels of desmethyl-clomipramine in CYP2D6 IMs and PMs (Stephan et al., 2006; Brown et al., 2017). Potentially, the CYP2D6 IM phenotype augmented the higher elevated concentration of desmethyl-clomipramine, produced from the rapid CYP2C19 enzymatic activity, leading to the reported ADRs.

#### 3.3.3 B18 and B46 (*CYP2D6*-Venlafaxine-Ineffectiveness pair)

B18, a CYP2D6 IM and CYP2C19 NM. B46, a CYP2D6 IM and CYP2C19 RM. Venlafaxine is mainly oxidized to O-desmethylvenlafaxine by CYP2D6 (Otton et al., 1996). Both venlafaxine and O-desmethylvenlafaxine are equipotent, but it has been suggested that the antidepressant effect of venlafaxine is largely accounted for by its metabolite (Lobello et al., 2010). The impaired CYP2D6 activity may reduce the formation of O-desmethylvenlaxine, thereby reducing the therapeutic effect.

### 3.4 Proportion of *CYP2D6/CYP2C19*-antidepressant-response pairs

Table 4 shows the proportion of actionable and non-actionable pairs for outcomes “antidepressant drug class,” “CPIC evidence level,” “*CYP2D6* and *CYP2C19* pharmacogenes,” and “drug response phenotype”.

**TABLE 4:** The proportion of actionable and non-actionable gene-antidepressant-response pairs.

Outcomes/Number (proportion)	Identified pairs	Actionable pairs (proportion, confidence interval)	Non-actionable pairs (proportion, confidence interval)
^a^Drug class (CPIC evidence level)			
All TCAs	25	12 (48%, 30–67)	13 (52%, 34–70)
*CYP2D6-*Nortriptyline (A)	13	5 (38%)	8 (62%)
*CYP2D6*-Amitriptyline (A)	3	2 (67%)	1 (33%)
*CYP2C19*-Amitriptyline (A)	3	3 (100%)	0 (0%)
*CYP2D6-*Clomipramine (B)	3	1 (33%)	2 (67%)
*CYP2C19-*Clomipramine (B)	3	1 (33%)	2 (67%)
All SSRIs	38	12 (32%, 19–48)	26 (68%, 53–81)
*CYP2D6-*Paroxetine (A)	2	1 (50%)	1 (50%)
*CYP2C19-*Sertraline (B)	15	3 (20%)	12 (80%)
*CYP2C19-*Citalopram (A)	6	3 (50%)	3 (50%)
*CYP2C19-*Escitalopram (A)	15	5 (33%)	10 (67%)
SNRIs	16	6 (38%, 18–61)	10 (62%, 39–82)
*CYP2D6*-Venlafaxine (A/B)	16	6 (38%)	10 (62%)
CPIC evidence level			
A	42	19 (45%, 31–60)	23 (55%, 40–69)
B	21	5 (24%, 11–45)	16 (76%, 55–90)
A/B	16	6 (38%, 18–61)	10 (62%, 39–82)
Pharmacogene			
*CYP2D6*	37	15 (41%, 26–57)	22 (60%, 43–74)
*CYP2C19*	42	15 (36%, 23–51)	27 (64%, 49–77)
Drug response phenotype			
Adverse reactions	50	24 (48%, 35–61)	26 (52%, 39–65)
Ineffectiveness	29	6 (21%, 10–38)	23 (79%, 62–90)

^a^ TCAs: tricyclic antidepressants, SSRIs: selective serotonin reuptake inhibitors, SNRIs: selective norepinephrine reuptake inhibitors.

Of the 79 *CYP2D6/CYP2C19*-antidepressant-response pairs, the pharmacogenetics of *CYP2D6* and *CYP2C19* was potentially able to explain a total of 30 (38%) pairs, making them actionable. A decreasing trend was observed for outcome ‘CPIC evidence levels’ from evidence level “A” (45%), “A/B” (38%) to “B” (24%). For “antidepressant drug class,” the proportion of actionable pairs across drug classes were 48% (TCAs), 38% (SNRIs), and 32% (SSRIs). Specifically, within TCAs-associated pairs, all *CYP2C19*-amitriptyline-response pairs (100%) were actionable, while the highest actionable proportion of SSRIs-associated pairs were *CYP2D6*-paroxetine-response pair (50%) and *CYP2C19*-citalopram-response pair (50%).

The actionability of *CYP2D6* and *CYP2C19* was comparable, up to 40% of the identified pairs were associated with the genotypes of the respective pharmacogene. Of the 50 *CYP2D6/CYP2C19*-antidepressant-ADRs pairs observed, up to 50% were actionable, while that of ineffectiveness pairs was lower, approximately 20%.

## 4 Discussion

### 4.1 Distribution of CYP2D6 and CYP2C19 genotype-inferred phenotypes

Among the 52 cases which experienced ADRs or ineffectiveness with the use of antidepressants, there were only eight cases (15%) with CYP2D6 and CYP2C19 NM phenotypes. This proportion is comparable to a recent study (∼13%) by Hahn and Roll (2022), who carried out a retrospective pharmacogenetic analysis in 108 European (German) adult depressive patients, where 51 of them were prescribed antidepressants or antipsychotics with CPIC and/or Dutch Pharmacogenetics Working Group (DPWG) guideline recommendations for *CYP2D6* and *CYP2C19* (Hahn and Roll, 2022). Hahn and Roll (2022) evaluated the clinical utility of *CYP2D6* and *CYP2C19* pharmacogenetics by comparing the proportion of actionable genotypes (genotypes with recommendations other than ‘initiate or treat with standard dose’) before and after pharmacogenetic testing service (Hahn and Roll, 2022). Separately, in a large Danish population-based case cohort of patients with mental disorders including depression (N = 51,464), 27% of the cases were reported with CYP2D6 and CYP2C19 NM phenotypes (Lunenburg et al., 2021). In addition to the larger sample size, the case cohort of this study also included patients with other mental disorders (e.g., bipolar disorder and schizophrenia), which may have explained the higher frequency observed. Despite the discordance, both the wider literature and our study showed that approximately 73%–85% of patients with mental disorders carry non-NM phenotypes in CYP2D6 and/or CYP2C19.

In the subgroup 45 cases used for *CYP2D6/CYP2C19*-antidepressant-response pair analysis, 57% were CYP2D6 non-NMs and 62% were CYP2C19 non-NMs. This is in accordance with Maggo et al. (2019a), who also reported comparably high proportions (42.7% CYP2D6 and 64% CYP2C19 non-NMs) in a cohort of Europeans and/or New Zealand Europeans with intolerance towards the use of SSRIs or SNRIs (Maggo et al., 2019a). However, the proportions of *CYP2D6* and *CYP2C19* actionable genotypes reported in Hahn and Roll (2022) were only 17% and 37%, respectively (Hahn and Roll, 2022). In addition to different study designs, the lower observation is likely due to the different definitions adopted for “actionable,”, which mainly concerns the “intermediate” metabolizer phenotype. For CPIC guidelines on antidepressants, not all IM phenotypes required dosing adjustments, the recommendation criterion which defined actionability in Hahn and Roll (2022). Taking amitriptyline as an example, CYP2D6 IMs are actionable, but not CYP2C19 IMs (Hicks et al., 2017). Unlike Hahn and Roll (2022), we consider all phenotypes in our cohort, apart from NMs, to potentially predispose to the risk of untoward drug responses. With external effects such as drug-drug interactions, IMs may be just as likely as PMs to experience ADRs, when compared with NMs. A recent systematic review suggested that IMs are more susceptible to phenoconversion associated with the concurrent administration of CYPs inhibitors, than other phenotypes (Klomp et al., 2020). With this assumption, the proportions reported by our study were consistent with other literature which observed approximately 20%–60% of depressed patients receiving psychotropic prescriptions potentially discordant with their pharmacogenetic profiles. However, these studies also included other pharmacogenes (e.g., *CYP1A2*, *CYP2C9*, and *CYP3A4/5*) (Hall-Flavin et al., 2012; Torrellas et al., 2017).

### 4.2 Explanatory value of *CYP2D6* and *CYP2C19* pharmacogenetics and the clinical implications

In this retrospective cohort reporting on ADRs or ineffectiveness with the use of antidepressants for mental health disorders, the majority of cases (∼60%) had actionable genotype-predicted *CYP2D6* and/or *CYP2C19* genotypes. When considering the type of responses, 48% of *CYP2D6/CYP2C19*-antidepressant-ADRs pairs and 21% of *CYP2D6/CYP2C19*-antidepressant-ineffectiveness pairs with CPIC evidence levels of A, A/B, or B had actionable pharmacogenetic information. This also means that for every 10 depressed patients presenting with ADRs and/or ineffectiveness, six patients would be expected to have a non-NM phenotype for either *CYP2D6* or *CYP2C19* or both. Furthermore, screening of these genotypes may potentially mitigate or prevent up to half of ADRs and one-fifth of ineffectiveness. This is a proportion considered to be of clinical significance for both prescriber and the patient.

Antidepressant response is a polygenic trait and has been studied using genome-wide association analyses (Tansey et al., 2013; Pain et al., 2021). While no clear association between antidepressant response and the pharmacogenetics of *CYP2D6* and *CYP2C19* were observed (Hicks et al., 2015; Hicks et al., 2017; Pain et al., 2021), the impact of *CYP2D6* and *CYP2C19* genetic variations on the variability in the metabolism of antidepressants is well-established with strong clinical implications (Carvalho Henriques et al., 2020). An early systematic review examined the pharmacokinetic influences of CYP2D6 and CYP2C19 genotype-inferred phenotypes on 20 antidepressants, expressed as percentages of dose adjustment (Kirchheiner et al., 2004). A good concordance between studies with respect to the dosing of TCAs was observed, which suggested halving the average TCAs doses in CYP2D6 PMs. This apparent association between the genetic variants of *CYP2D6* and *CYP2C19* and the pharmacokinetics of TCAs may have explained the high proportion of actionable *CYP2D6/CYP2C19*-TCAs-response pairs, as reported in our cohort.

Our findings from this real-world case series highlights the predictive value of *CYP2D6* and *CYP2C19* pharmacogenetics. This is supported by literature where the genotypes of either *CYP2D6* and *CYP2C19* were associated with the efficacy and tolerability profile of antidepressants (Shams et al., 2006; Penas-Lledo et al., 2013; He et al., 2017; Fabbri et al., 2018; Jukic et al., 2018; Zastrozhin et al., 2021; Campos et al., 2022; Jokovic et al., 2022; Thiele et al., 2022). However, there were also negative and mixed findings reported (Brandl et al., 2014; Hodgson et al., 2015; Taranu et al., 2017; Maggo et al., 2019a). Notwithstanding this, the predictive role of *CYP2D6* and *CYP2C19* pharmacogenetics in antidepressant response was substantiated in recent systematic reviews and meta-analyses (Arnone et al., 2023; Brown et al., 2022; Solomon et al., 2019).

By individualizing our analysis for each reported response, this case series showed that pharmacogenetics is associated with the efficacy profile (∼20%) of antidepressants to a lesser extent than their tolerability (∼50%). Mrazek et al. (2011) investigated the role of *CYP2D6* and *CYP2C19* genetic variants in citalopram response, in 1,074 White non-Hispanic subjects, previously enrolled in the STAR*D trial. They reported that the CYP2C19*2 allele was significantly associated with a lower tolerability (*p* = 0.02), but not remission rate (*p* = 0.95) (Mrazek et al., 2011). Pharmacodynamic aspects such as variation of genes involved in antidepressant response (Bahramali et al., 2016; Firouzabadi et al., 2017; van Westrhenen et al., 2020), and the functional selectivity and intrinsic efficacy of a drug (Berg and Clarke, 2018), may have contributed to the heterogenous ineffectiveness phenotypes. Furthermore, commonly prescribed antidepressants such as sertraline, citalopram, escitalopram and venlafaxine have a wide therapeutic window (Marken and Munro, 2000; Hansen et al., 2017), where the wide range between minimum effective and minimum toxic concentrations makes the efficacy profile of antidepressants less susceptible to a change in the drug exposure level induced by pharmacogenetics.

For ADRs, 48% of *CYP2D6/CYP2C19*-antidepressant-response pairs in our cohort were actionable. In comparison, the recent PREPARE trial that showed a reduction in ADRs stemming from genotype-guided prescribing, reported that of those specific antidepressants that were prescribed to at least 10 of a total of 6,944 enrolled patients, actionable *CYP2D6/CYP2C19* variants were present in 25% (nortriptyline) to 47% (venlafaxine) (Swen et al., 2023). There are two possible reasons for the lower actionability observed in the PREPARE trial. First, the PREPARE participants were patients embarking on drug therapy, whereas our cohort included patients who had already started and experienced ADRs, which are therefore a more select group. Second, the PREPARE trial adopted DPWG guidelines for all phenotyping and actionability assignment, while for our study, we worked mainly on CPIC guidelines. However, this also means that almost half of the pairs in our study were not associated with the genotypes of *CYP2D6* and/or *CYP2C19*, highlighting the role of external factors. Campos et al. (2021) applied a polygenic risk score approach to study common ADRs reported with the use of antidepressants (e.g., weight gain, suicidality, and sexual dysfunction). The significant associations observed were antidepressant- and ADR-dependent. For example, body mass index was strongly associated with weight gain across different antidepressant drug class, while headache showed significance with the use of sertraline only. However, this study also observed a high likelihood for a participant to report the same ADRs across different antidepressants, indicating the presence of an unknown common ‘element’ which may be a result of pharmacological and genetic factors (Campos et al., 2021). To add complexity, evidence suggesting pharmacogene-dependent ADRs is emerging. Eugene (2019) extracted a total of 5,000 post-marketing SSRIs-associated ADRs cases, and by determining the drugs as CYP2D6 or CYP2C19 substrates, clustered these cases into two groups. This study observed a differential nature of ADRs exhibited by CYP2D6 and CYP2C19 SSRIs substrates, with the latter mostly associated with the modulation of autonomic nervous system (Eugene, 2019).

Apart from a patient’s pharmacogenetic profile, other aspects such as drug-drug interactions and phenoconversion should be considered during regime optimization. In a cohort of 60 patients taking antidepressants, Gloor et al. (2022) compared the intrinsic (genotypic) and observed (phenotypic) activity of six cytochrome enzymes: CYP1A2, 2B6, 2C9, 2C19, 2D6, 3A4, and 3A5 (Gloor et al., 2022). They observed a consistently lower than predicted enzymatic activity for CYP2D6, CYP2C19, and CYP2C9 (p < 8 × 10^−3^ for all observations). The clinical impact of phenoconversion is increasingly recognized (Cicali and Wiisanen, 2022; Mostafa et al., 2022; Nahid and Johnson, 2022), with different approaches designed to incorporate this factor for efficient genotype-to-phenotype prediction (Cicali et al., 2021). While phenoconversion is multi-factorial, the proportion of patients taking antidepressants at risk of experiencing ADRs or ineffectiveness may be higher than is expected from their genotypes, highlighting the importance of pharmacogenetic screening.

## 5 Limitations

There are several limitations in the current study. First, this is a small clinical cohort (N = 52). Second, the degree of association between the genotype-inferred phenotypes, drug exposure, and drug response events, used in determining the actionability for gene-antidepressant-response pairs, remains indefinite. In addition to having a polygenic complex trait, the presentation of drug response is heterogenous, as observed with the ineffectiveness phenotypes. In this study, separate analysis for these phenotypes was limited by the small sample size. Potentially, the observed response event may partly be accounted for by other factors, which may have an impact on the association, yet, were unknown at the time of recruitment.

A third limitation concerns the selective genotyping of *CYP2D6* and *CYP2C19*, where screening for other CYPs, transporters or pharmacodynamic pharmacogenes relevant to antidepressant response may be useful. Specifically, for *CYP2C19*, the selective allelic screening may have missed novel or other clinically significant variants. Fourth, healthcare practitioners (referrer) or patient (self-referral) were asked to provide medical histories, which in part, were recall dependent. Thus, the risk of recall bias remains. Fifth, there were several cases with incomplete clinical information (e.g., the specific type, dosing, and frequency of antidepressant(s) associated with the reported ADRs or ineffectiveness, and event description), thus limiting analysis. Sixth, the lack of pharmacokinetic data. Collecting and measuring drug levels from appropriate serum or plasma samples will be useful in elucidating the association between genotypes and drug exposure.

## 6 Future work and conclusion

There are several suggestions for future research. First, prioritizing referral cases from healthcare practitioners for pharmacogenetic analysis. Since antidepressant response is highly subjective, this approach may ensure that the reported responses are validated against clinical practice guidelines. Second, evaluating the clinical utility of *CYP2D6* and *CYP2C19* pharmacogenetics *via* further collaboration with clinical colleagues. For example, how did the pharmacogenetic results inform their subsequent prescribing and management of the patient? Third, encouraging more complete clinical information from the referrers. Fourth, stratifying and analysing cases according to drug class to minimize case heterogeneity. However, this would require a very large sample size. In regards to antidepressant responses, a combinatorial approach may be helpful in understanding the comprehensive impacts of pharmacogenetics. For example, the metabolism of sertraline involves CYP2D6, CYP2C19, CYP2C9, CYP2B6, and CYP3A4. However, apart from *CYP2D6* and *CYP2C19* to a lesser extent, the functional characterization of variants of other pharmacogenes remains uncertain, thus limiting the assignment of CPIC evidence levels.

In conclusion, this retrospective cohort describes 52 mental health cases who experienced ADRs or ineffectiveness or both with antidepressants. 79 *CYP2D6/CYP2C19*-antidepressant-response pairs with CPIC evidence level of A, A/B, or B were identified. Using CPIC guidelines, approximately 50% ADRs-associated pairs and 20% ineffectiveness-associated pairs were actionable with the pharmacogenetics of *CYP2D6* and *CYP2C19*. With this, we provide an insight into the clinical utility of *CYP2D6* and *CYP2C19* in antidepressant prescribing. It is important to note that all pharmacogenetic data is valuable, regardless of its “actionability,” as it facilitates prescribers in initiating, maintaining, or adjusting antidepressant therapy.

## Data Availability

The original contributions presented in the study are included in the article/Supplementary Material, further inquiries can be directed to the corresponding author.

## Abbreviations

ADRs, adverse reactions; CPIC, clinical pharmacogenetics implementation consortium; CYP, Cytochrome P450; DNA, deoxyribonucleic acid; DPWG, dutch pharmacogenetics working group; IM, intermediate metabolizer; MAO-I, monoamine oxidase inhibitors; NM, normal metabolizer; Pharmvar, pharmacogene variation consortium; PCR, polymerase chain reaction; PM, poor metabolizer; RM, rapid metabolizer; SNRIs, selective norepinephrine reuptake inhibitors; SSRIs, selective serotonin reuptake inhibitors; STAR*D, sequenced treatment alternatives to relieve depression; TCAs, tricyclic antidepressants; UDRUGS, understanding adverse drug reactions using genomic sequencing; UM, ultrarapid metabolizer.
