# Supplementary material for: The pharmacogenetics of CYP2D6 and CYP2C19 in a case series of antidepressant responses
Source: Front Pharmacol. 2023 Feb 21;14:1080117. doi: 10.3389/fphar.2023.1080117 (PMC9988947; doi:10.3389/fphar.2023.1080117)
Supplement: Supplementary file 2 [file Table1.docx]

*Supplementary Table 1 List of CYP2D6 or CYP2C19-antidepressant pairs with CPIC evidence level A, A/B, or B.*

| **Drugs/*Genes*** | ***CYP2D6*** | ***CYP2C19*** |
| --- | --- | --- |
| Amitriptyline | A | A |
| Citalopram | N/A | A |
| Escitalopram | N/A | A |
| Nortriptyline | A | N/A |
| Paroxetine | A | N/A |
| Venlafaxine | A/B | N/A |
| Vortioxetine | A/B | N/A |
| Clomipramine | B | B |
| Desipramine | B | N/A |
| Doxepin | B | B |
| Fluvoxamine | B | N/A |
| Imipramine | B | B |
| Sertraline | C | B |
| Trimipramine | B | B |

Data retrieved from the Clinical Pharmacogenetics Implementation Consortium website at <https://cpicpgx.org/genes-drugs/>; accessed [26^th^ January 2023]; updated [1^st^ June 2022].
