# Supplementary figures and images for: The pharmacogenetics of CYP2D6 and CYP2C19 in a case series of antidepressant responses
Source: Front Pharmacol. 2023 Feb 21;14:1080117. doi: 10.3389/fphar.2023.1080117 (PMC9988947; doi:10.3389/fphar.2023.1080117)

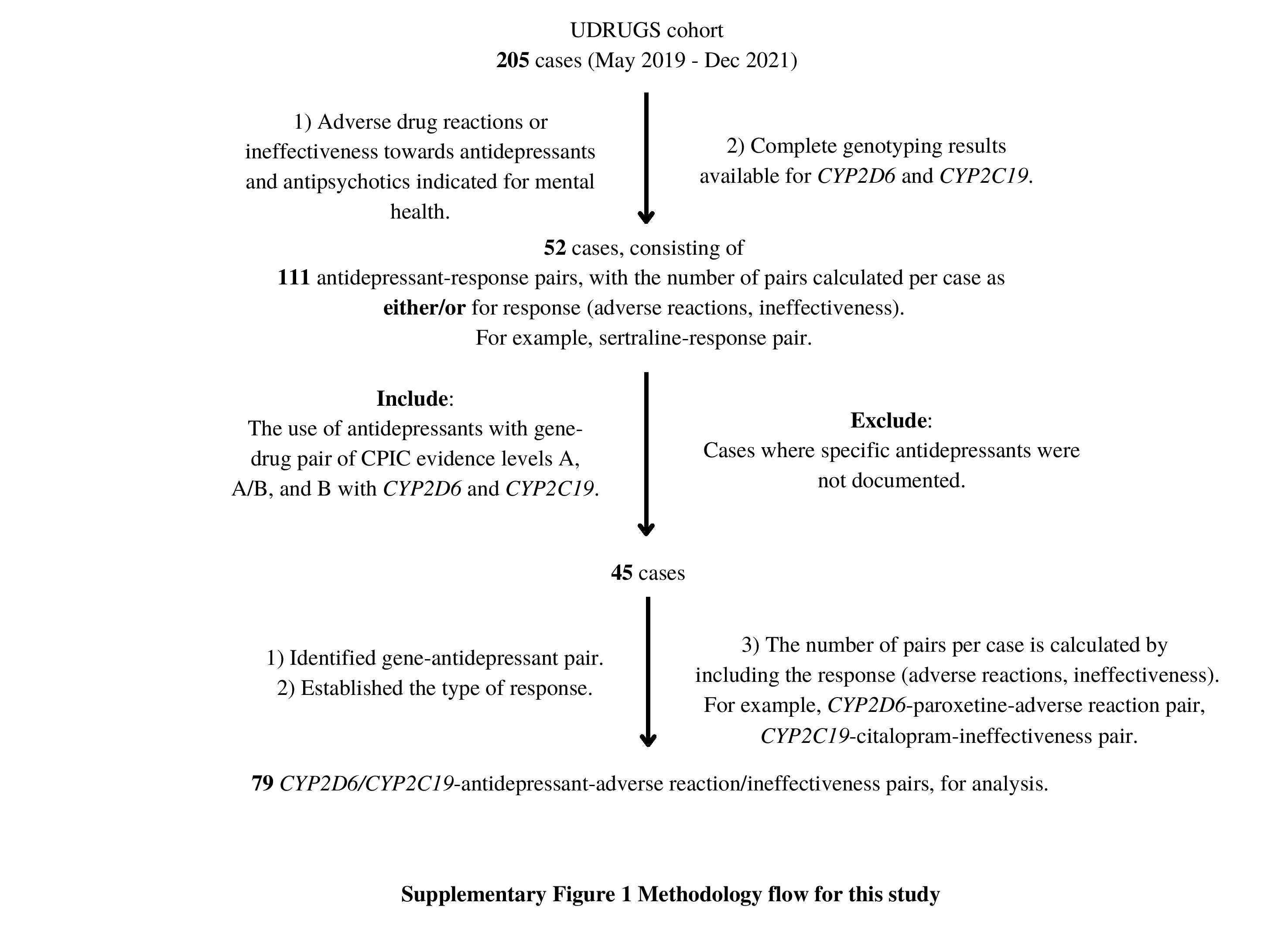

Supplement: Supplementary file 3 [file Image1.jpg]
